# Luteolin reduces inflammation in *Staphylococcus aureus*-induced mastitis by inhibiting NF-κB activation and MMPs expression

**DOI:** 10.18632/oncotarget.16092

**Published:** 2017-03-10

**Authors:** Ying-fang Guo, Nian-nian Xu, Weijing Sun, Yifan Zhao, Cheng-ye Li, Meng-yao Guo

**Affiliations:** ^1^ College of Veterinary Medicine, Huazhong Agricultural University, Wuhan 430070, People's Republic of China

**Keywords:** luteolin, mastitis, Staphylococcus aureus (S. aureus), anti-inflammation, inflammatory signal pathway

## Abstract

Mastitis is a serious and prevalent disease caused by infection by pathogens such as *Staphylococcus aureus*. We evaluated the anti-inflammatory effects and mechanism of luteolin, a natural flavonoid with a wide range of pharmacological activities, in a mouse model of S. *aureus* mastitis. We also treated cultured mouse mammary epithelial cells (mMECs) with S. *aureus* and luteolin. Histopathological changes were examined by H&E staining and the levels of inflammatory cytokine proteins were analyzed using ELISAs. We determined mRNA levels with qPCR and the level of NF-κB and matrix metalloproteinase (MMP) proteins by Western blotting. The observed histopathological changes showed that luteolin protected mammary glands with S. *aureus* infection from tissue destruction and inflammatory cell infiltration. Luteolin inhibited the expression of TNF-α, IL-1β, and IL-6, all of which were increased with S. *aureus* infection of mammary tissues and mMECs. S. *aureus*-induced TLR2 and TLR4 was suppressed by luteolin, as were levels of IκBα and NF-κB p65 phosphorylation and expression of MMP-2 and MMP-9. Levels of tissue inhibitor of metalloproteinases (TIMP)-1 and TIMP-2 were enhanced. These findings suggest luteolin is a potentially effective new treatment to reduce tissue damage and inflammation from S. *aureus*-induced mastitis.

## INTRODUCTION

Mastitis, initially defined as an inflammatory response of the mammary glands caused by invading bacteria, is a serious disease for both humans and animals [1]. *Staphylococcus aureus (S. aureus)* is a gram-positive bacterium which has been recognized as a major pathogen infecting a range of hosts [2, 3] and has been employed in previous studies which used a mouse model of microbial mastitis [4]. However, there is still no effective treatment for *S. aureus*-induced mastitis.

Local innate immunity plays a key role in initiating and coordinating homeostasis and resistance to intramammary infection by regulating effector cytokines and other mediators of inflammation [5]. A previous study showed that mouse mammary epithelial cell (mMECs) recognition of the infection through the activation of several pattern recognition receptors (PRRs) is necessary for the initiation of the immune response in the mammary glands [6]. Once *S. aureus* infects the mammary glands, these receptors induce innate immune by producing mediators of inflammation and local defense [7]. Toll-like receptors (TLR), a type of PRR receptor, were identified as pivotal immune receptors [8]. TLR-2 is activated by several classes of microorganisms such as peptidoglycan and lipoteichoic acid, which are major components of the cell wall pathogen-associated molecular pattern (PAMP) of Gram-positive bacteria, including *S. aureus* [9]. It has been reported that TLR-4 also plays an important role in the *S. aureus* inflammation process [10]. In TLR-2 and TLR-4 signaling, PAMP recognition by TLRs leads to the activation of downstream signaling molecules such as nuclear factor-kappa B (NF-κB). NF-κB promotes expression of target genes that mediate expression of inflammatory cytokines, such as tumor necrosis factor-α (TNF-α), interleukin-1β (IL-1β), and IL-6 [11, 12]. NF-κB and inflammatory cytokines regulate the expression of matrix metalloproteinases (MMPs) and its inhibitor tissue inhibitor of metalloproteinases (TIMPs) [13, 14]. Increased expression of MMPs has been associated with the inflammatory process [15].

**Figure 1:**
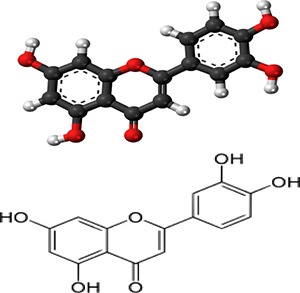
Chemical structure of luteolin

Luteolin (Figure 1) is a member of the flavone subclass of flavonoids, found in many vegetables, fruits, and tea [16]. Varied pharmacological activities of luteolin have been observed, including antioxidant, anti-mutagenic, anti-inflammatory, and anti-allergic [17–19]. Some studies have reported that luteolin profoundly reduces lipopolysaccharide (LPS)-induced inflammation, efficiently inhibits the LPS-induced pro-inflammatory molecule expression *in vitro*, and reduces leukocyte infiltration in tissue [20]. The present study was performed to identify the effect of luteolin on the inflammatory response in the mouse model of *S. aureus* mastitis and to analyze the underlying mechanisms of its effect.

## RESULTS

### In vivo

### Histopathological changes

**Figure 2:**
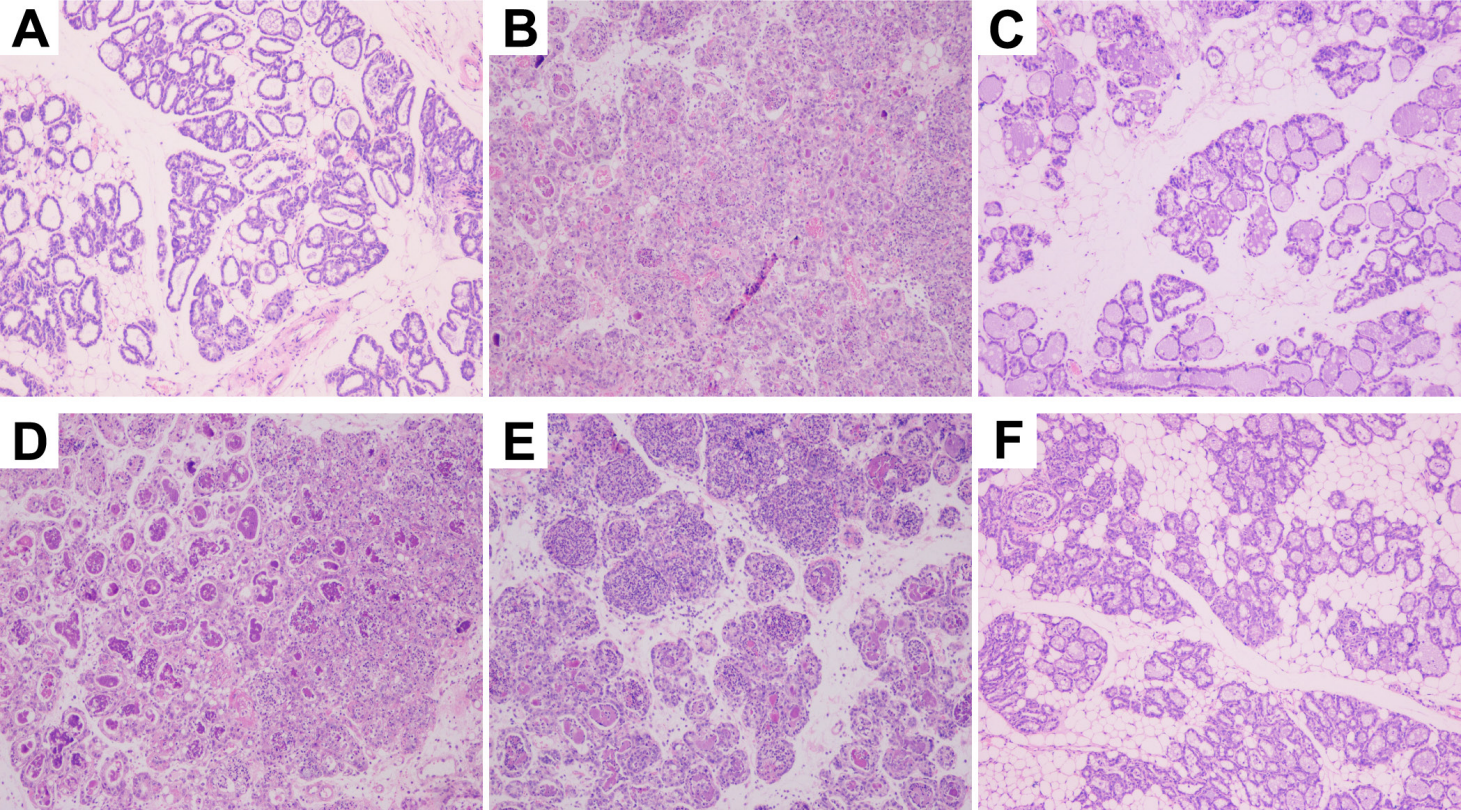
Histopathology of mammary gland tissues (**A**) Mammary gland tissue of control group, (**B**) *S. aureus* group, (**C**) *S. aureus* + Dex group, (**D–F**) luteolin treatment groups, administered 25, 50, and 100 mg/kg luteolin.

Mammary gland tissues from all groups were harvested 24 h after *S. aureus* inoculation and luteolin treatment, cut into sections, and H&E stained. Normal structure and no histopathological lesions were observed in the BCG group (Figure 2A). In the *S. aureus* group (without luteolin treatment) (Figure 2B), many of the mammary epithelial cells were destroyed, and the organizational structure of the mammary lobules was incomplete. Inflammatory cells including neutrophils and macrophages were observed in the mammary tissues. However, luteolin inhibited the pathological damage caused by *S. aureus*. In the LAG group, the inflammatory cell infiltration was decreased and the acini and lobules were not destroyed. The histopathological changes were ameliorated in a dose-dependent manner at drug concentrations of 25, 50, and 100 mg/kg (Figure 2D, 2E, 2F). There were mild pathological lesions in the DEX group (Figure 2B).

### Effects of luteolin on inflammatory cytokine expression

**Figure 3:**
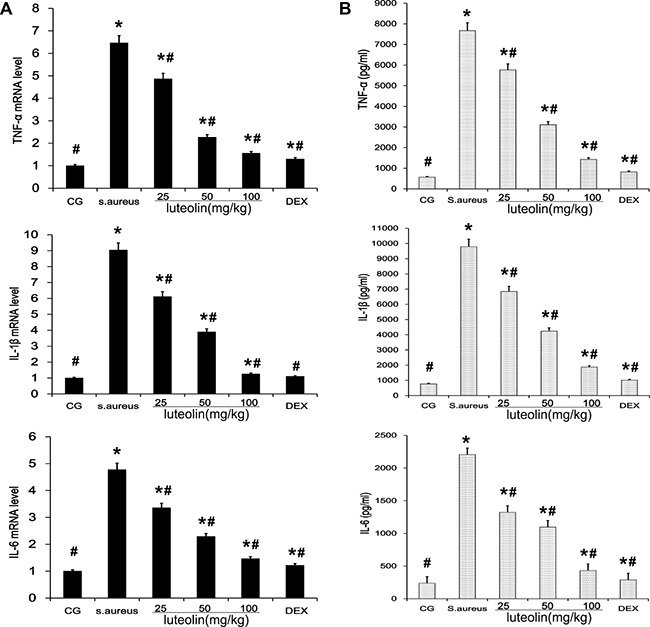
Effects of luteolin on cytokine expressions (**A**) The expression levels of cytokines TNF-α, IL-1β, and IL-6 induced by *S.aureus* in mammary gland tissues were determined by qPCR. (**B**) The protein levels of TNF-α, IL-1β, and IL-6 induced by *S.aureus* was detected by ELISA in mammary gland homogenates. *p < 0.05 indicates a significant difference from the CG; ^#^*p* < 0.05 indicates a significant difference from *S. aureus*.

*S*. aureus-induced mastitis is related to many types of inflammatory cytokines such as TNF-α, IL-1β and IL-6 [21]. The tissues were harvested 24 h after stimulation with *S. aureus*, and qPCR and ELISA analysis were used to evaluate the mRNA and protein levels of inflammatory cytokines in *S. aureus*-induced mammary gland. Compared with the BCG group, the TNF-α, IL-1β and IL-6 mRNA and protein levels in the *S. aureus* group were significantly increased. These increases were significantly inhibited by luteolin. These results indicated that luteolin downregulated *S. aureus*-induced elevation of TNF-α, IL-6, and IL-1β mRNA and proteins in a dose-dependent manner (Figure 3A and 3B).

### Effect of luteolin on TLR-2 and TLR-4 expression

**Figure 4:**
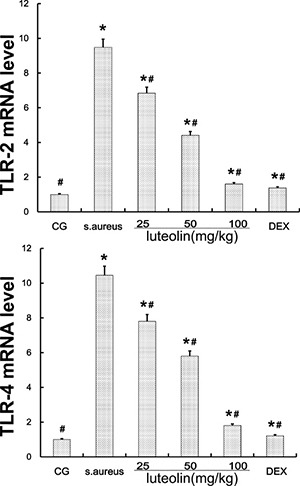
TLR-2 and TLR-4 expression in tissues The TLR-2 and TLR-4 mRNA levels were measured by q-PCR. *GAPDH* was used as a control. Data displayed represent the means ± S.D. *p < 0.05 indicates a significant difference from the CG; ^#^*p* < 0.05 indicates a significant difference from *S. aureus*.

TLR-2 and TLR-4 signaling, which play a vital role in the inflammatory response, were the major immune receptors involved in the regulation of *S. aureus*-induced inflammation. The *S. aureus* group had significantly higher TLR-2 and TLR-4 mRNA and protein levels than the BCG and LAG groups. Luteolin suppressed TLR-2 and TLR-4 mRNA and protein levels in a dose-dependent manner as the drug concentration was increased to 25, 50, and 100 mg/kg (Figure 4) in mammary tissue.

### Luteolin suppressed the activation of the NF-κB pathway in mammary tissues

**Figure 5:**
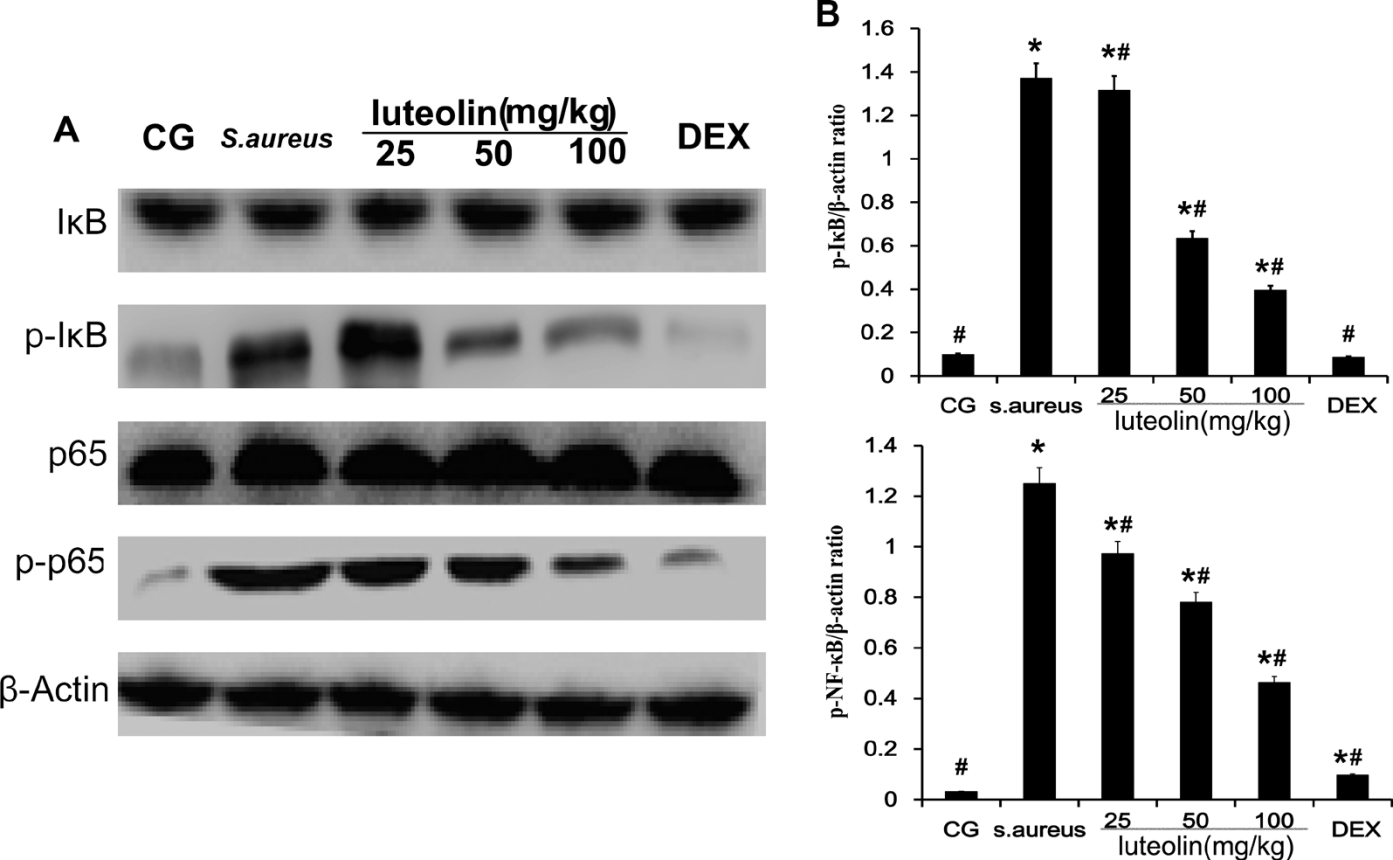
Effects of luteolin on NF-κB pathway The levels of p65 and IκBα protein in mammary gland tissues. Phosphorylation of p65 and IκBα were analyzed using phospho-specific anti-pp65, phospho-specific anti-pIκBα antibodies. β-actin was used as control. *p < 0.05 indicates a significant difference from the CG; ^#^*p* < 0.05 indicates a significant difference from *S. aureus*.

NF-κB plays a major role in promoting the expression of various pro-inflammatory cytokines in response to *S.aureus* [22]. Activation of NF-κB results in the phosphorylation and ubiquitination of the inhibitory subunit, IκB, and translocation of p50 and p65 from the cytoplasm to the nucleus [23]. In the present study, IκB and NF-κB protein levels were measured using Western blot analysis (Figure 5). Compared to the BCG, there was a significant increase (*P* < 0.05) in IκB and NF-κB protein levels in the *S. aureus* group, LAG group, and the DEX group. The *S. aureus* group exhibited significantly higher levels of IκBα and P65 phosphorylation than the BCG and LAG groups. However, luteolin significantly suppressed IκBα and P65 protein phosphorylation in a dose-dependent manner.

### Luteolin reduced levels of MMPs and increased TIMPs in mammary gland

**Figure 6:**
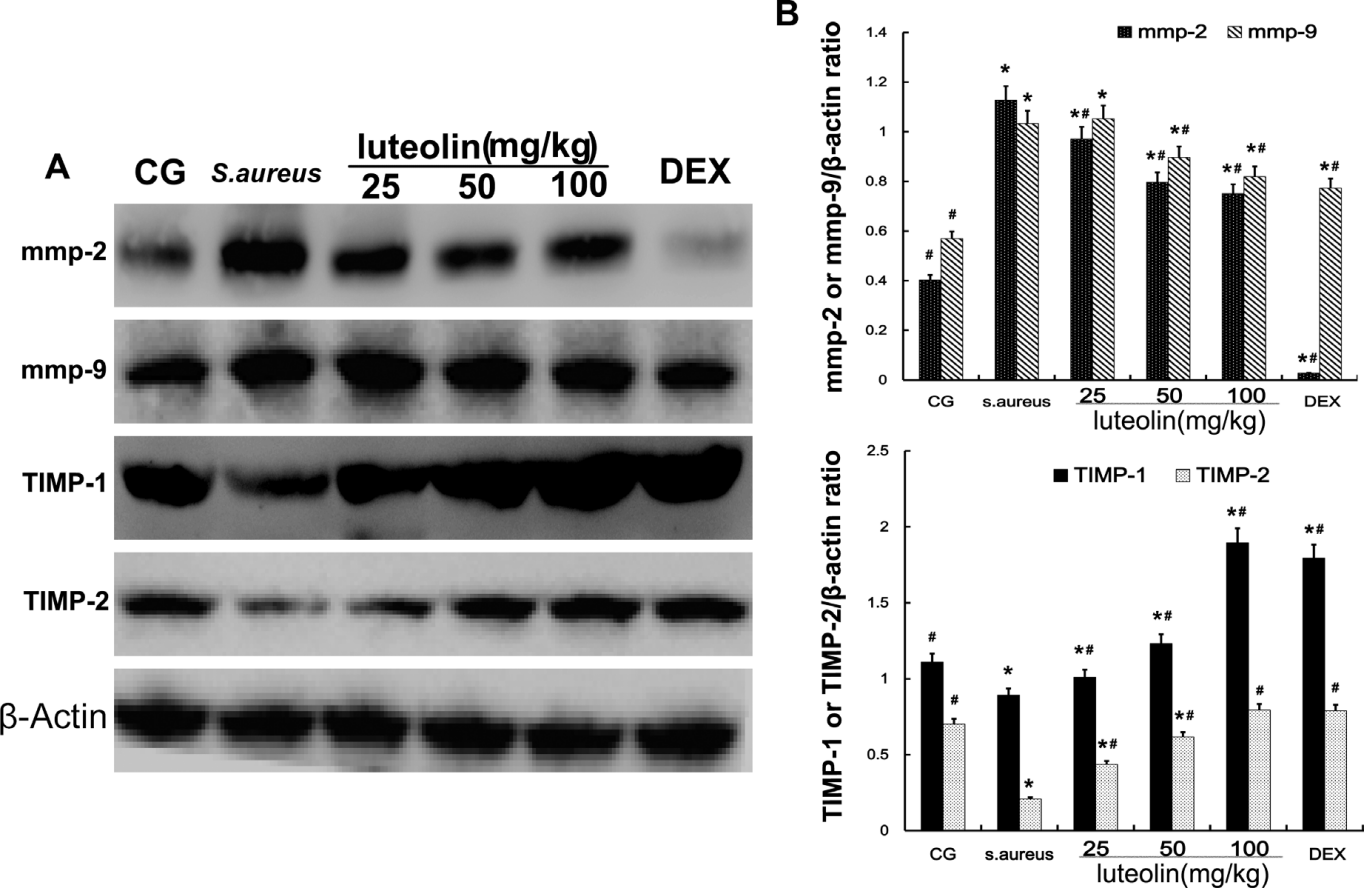
Effects of luteolin on the MMPs and TIMPs MMP-2, MMP-9, TIMP-1, and TIMP-2 were analyzed by Western blot. β-actin was used as control. The data displayed represent the means ± S.D. *p < 0.05 indicates a significant difference from the CG; ^#^*p* < 0.05 indicates a significant difference from *S. aureus*.

Gelatinase A (MMP-2) and gelatinase B (MMP-9) have an important pathological significance, closely related to the inflammatory process. Western blot analysis showed that the *S. aureus* group had significantly higher MMP-2 and MMP-9 levels than the BCG and LAG groups. The levels of MMP-2 and MMP-9 were inhibited by luteolin at concentrations of 25, 50, and 100mg/kg (Figure 6). At higher drug concentrations, the MMP-2 and MMP-9 levels were clearly decreased. TIMP-2 inhibits the activity of MMP-2 through direct binding and TIMP-1 inhibits MMP-9 [24, 25]. Compared to BCG, stimulation with *S. aureus* led to a significant increase in TIMP-1 and TIMP-2 expression. As the concentration of luteolin was increased to 25, 50, and 100 mg/kg, the level of TIMP-1 and TIMP-2 was increased in a dose-dependent manner (Figure 4).

### *In vitro* studies agreed with mammary gland tissue results

**Figure 7:**
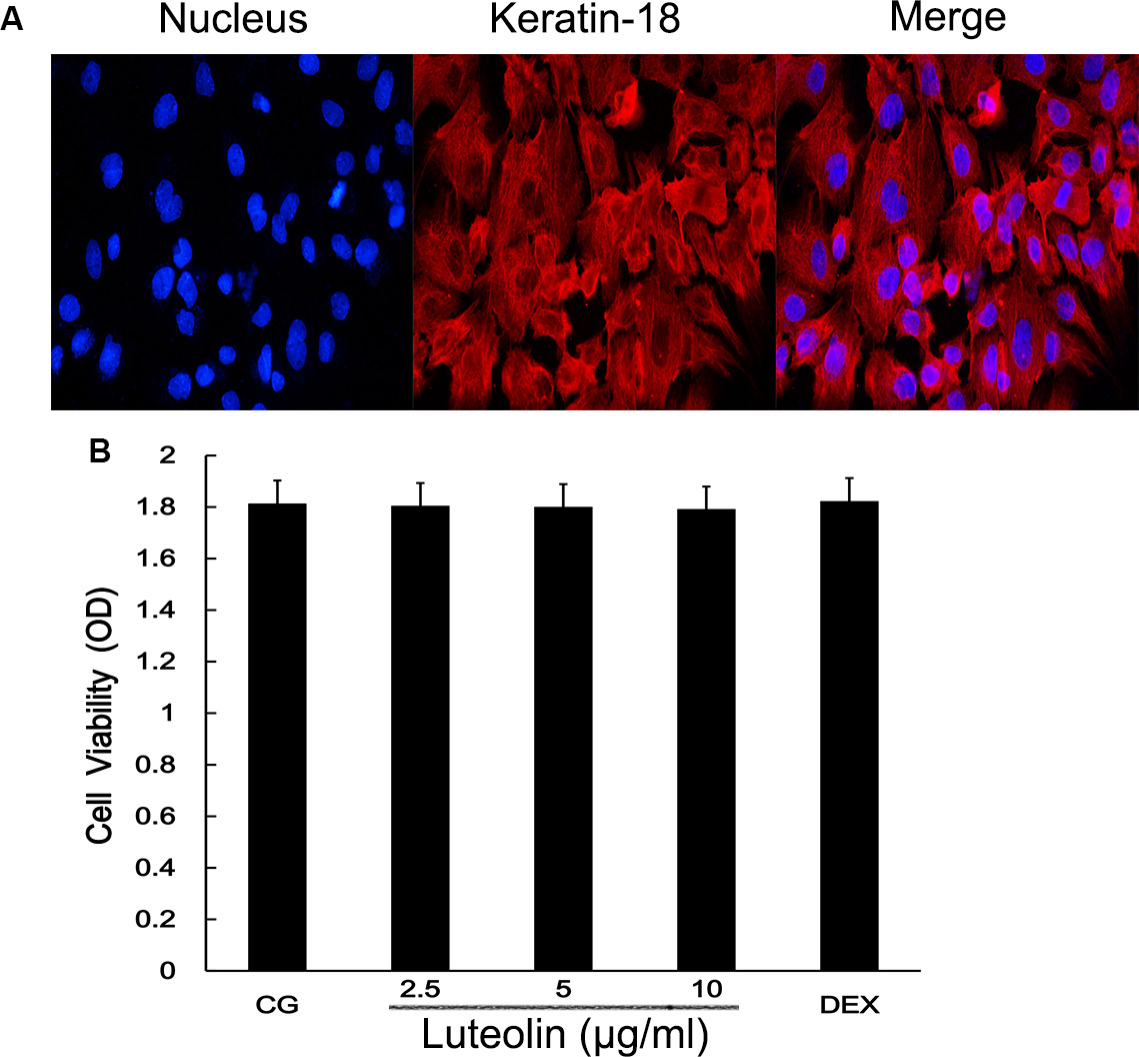
Mammary epithelial cell identification and cell viability assay (**A**) cells were pretreated with fluorochrome to observe the mammary gland cell integrity. (**B**) The potential cytotoxicity of luteolin (2.5, 5.0, and 10 μg/ml) was evaluated by the MTT assay. The cell nucleus is marked by blue fluorescence. Keratinose 18 is the green fluorophore.

We took the mouse mammary gland from pregnant mice and used enzymatic digestion to extract mouse mammary epithelial cells (mMECs). Cells were adhered for growth and microscopic examination revealed them to be pebble-shaped. After proliferation, cells integrated tightly and formed a dome-shaped structure. Keratin-18 was used to detect the mMECS (Figure 7A), green fluorescence indicated that cells were cytokeratin positive. Treatment with luteolin did not affect cell viability at any concentration (2.5, 5, and 10 μg/ml) (Figure 7B).

**Figure 8:**
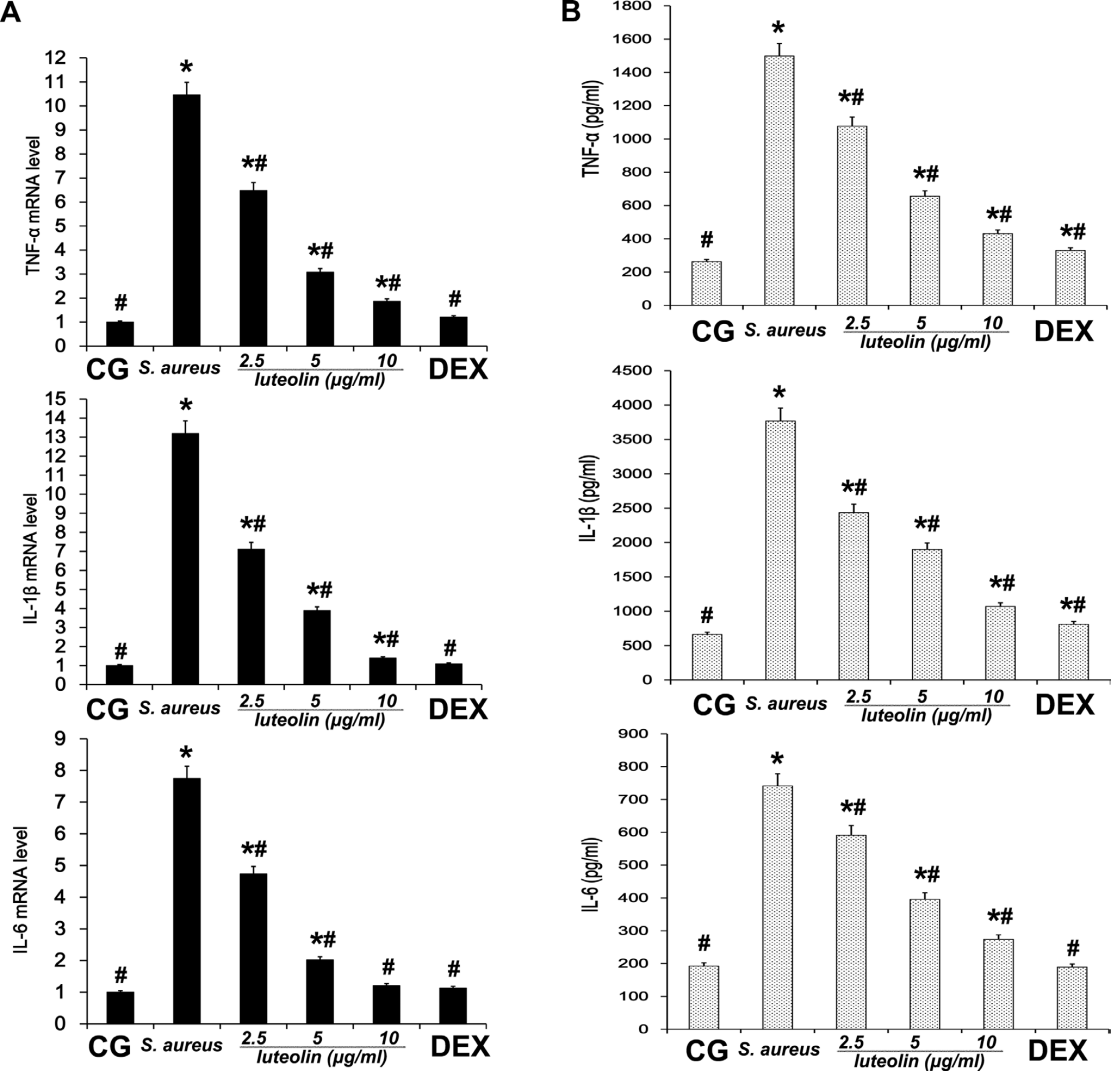
Effects of luteolin on cytokine expression in mammary epithelial cell (**A**) The expression levels of cytokines TNF-α, IL-1β, and IL-6 induced by *S.aureus* were determined by qPCR in cells. (**B**) The protein levels of TNF-α, IL-1β, and IL-6 induced by *S.aureus* were detected by ELISA in cells. *p < 0.05 indicates a significant difference from the CG; ^#^*p* < 0.05 indicates a significant difference from *S. aureus*.

**Figure 9:**
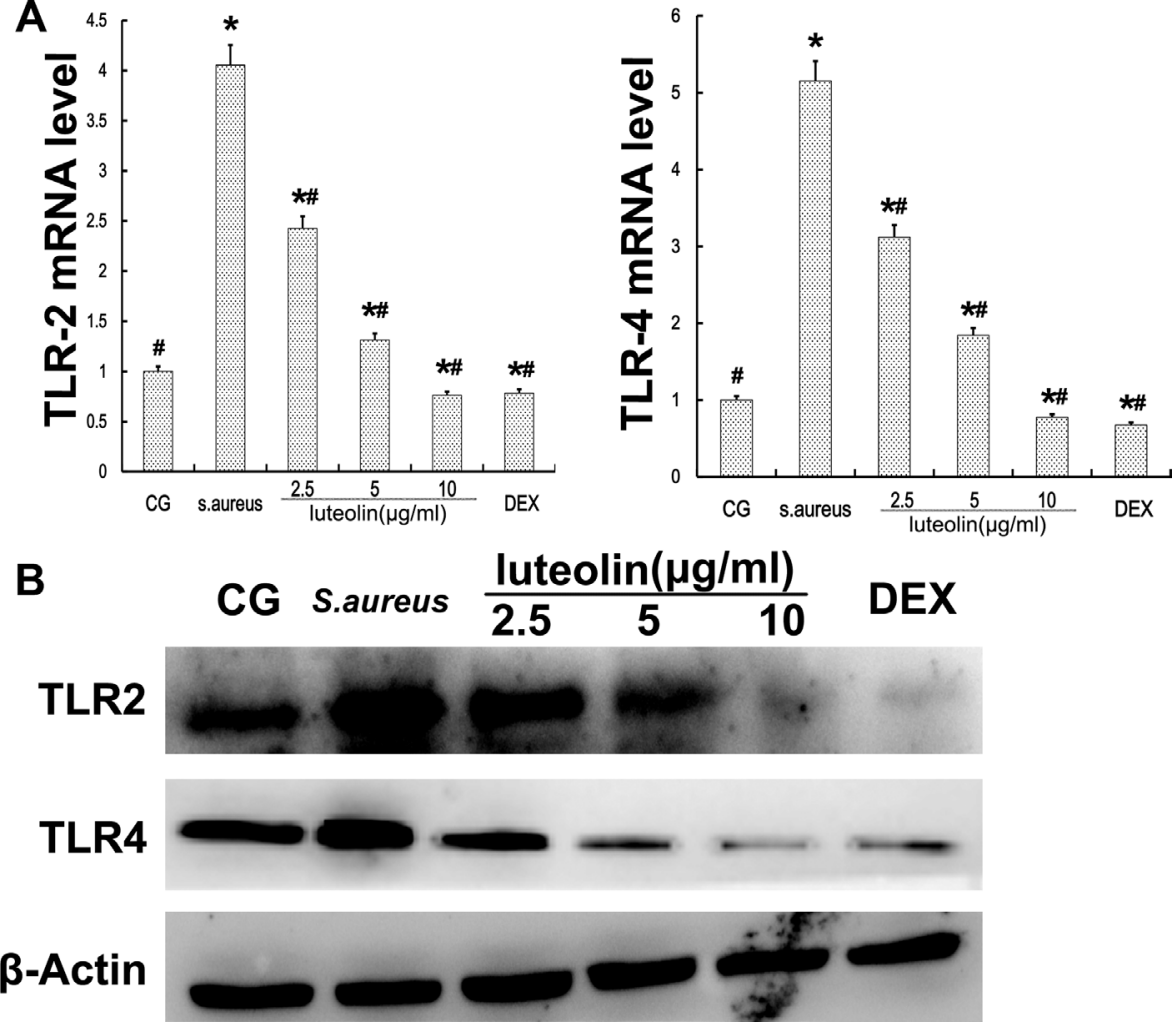
TLR-2 and TLR-4 expression in mammary epithelial cells (**A**) TLR-2 and TLR-4 mRNA levels were measured by q-PCR. (**B**) proteins were analyzed by Western blot. β-actin was used as control. *p < 0.05 indicates a significant difference from the CG; ^#^*p* < 0.05 indicates a significant difference from *S. aureus*.

**Figure 10:**
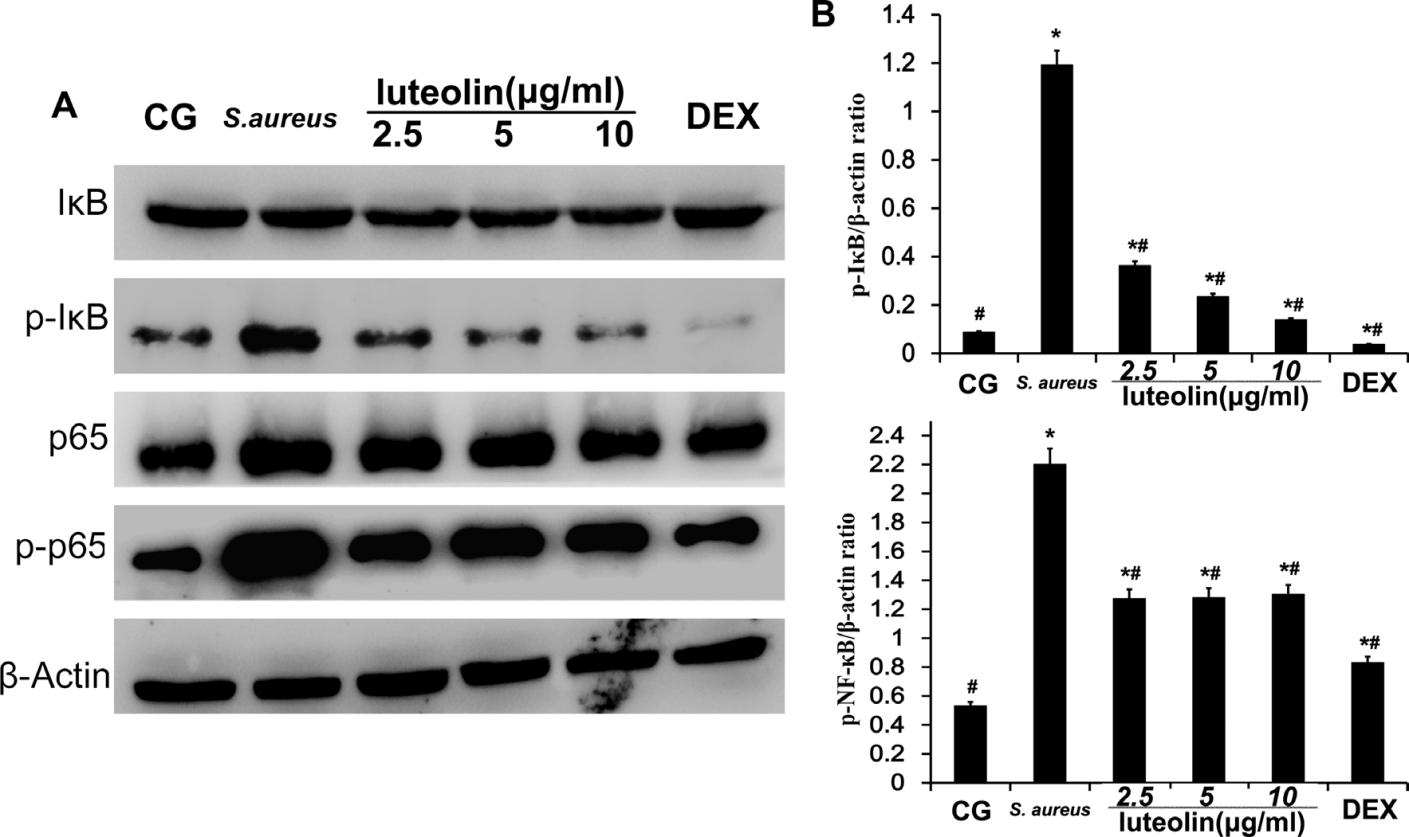
Effects of luteolin on the NF-κB pathway in mammary epithelial cells The levels of p65 and IKBα protein were analyzed by Western blot. Phosphorylation of p65 and IκBα were analyzed using phospho-specific anti-pp65 and phospho-specific anti-pIκBα antibodies. β-actin was used as control. *p < 0.05 indicates a significant difference from the CG; ^#^*p*<0.05 indicates a significant difference from *S. aureus*.

**Figure 11:**
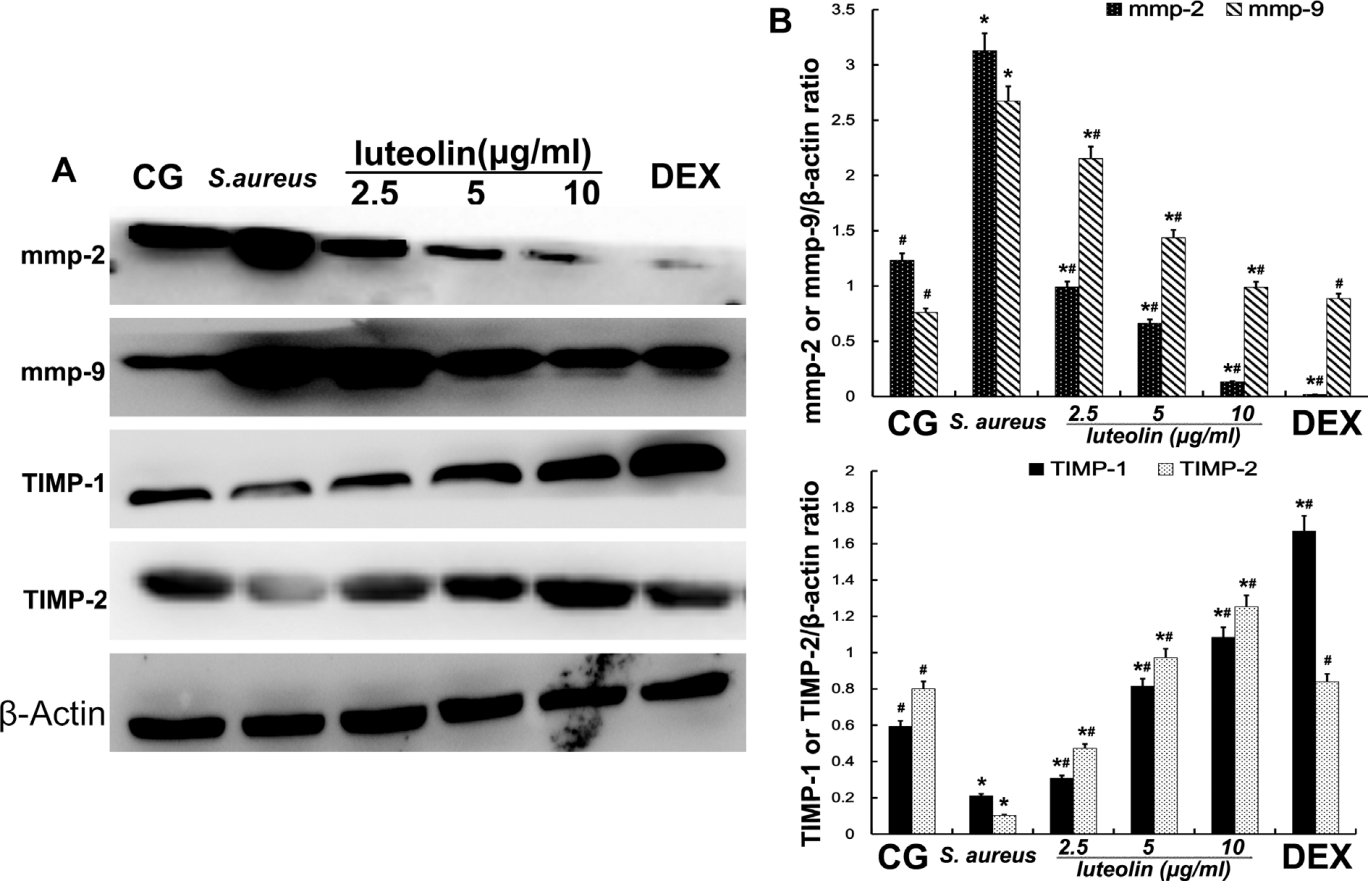
Effects of luteolin on the MMPs and TIMPs in mammary epithelial cells MMP-2, MMP-9, TIMP-1, and TIMP-2 were analyzed by Western blot. β-actin was used as control. The data displayed represent the means ± S.D. *p < 0.05 indicates a significant difference from the CG; ^#^*p* < 0.05 indicates a significant difference from *S. aureus*.

Cells were cultured in six-well plates in Dulbecco's Modified Eagle Medium (DMEM) containing FBS (10%) for 24 h. After 1 h of incubation with luteolin, *S.aureus* was added into the wells and incubated for 2 h. Then, the medium and cells were collected to examine the effect of luteolin on mRNA and protein levels of inflammatory proteins (TNF-α, IL-1β, IL-6) (Figure 8). As in the tissue experiments, luteolin reduced the TNF-α, IL-1β, IL-6, TLR-2 and TLR-4 mRNA and protein levels in a dose-dependent manner (Figure 9). Additionally, luteolin significantly suppressed IκBα and p65 protein phosphorylation (Figure 10). When the concentration of luteolin was increased to 2.5, 5, and 10 μg/ml, the levels of TIMP-1 and −2 were increased in a dose-dependent manner, while MMP-2, and −9 were decreased, in agreement with the tissue sample results (Figure 11).

## DISCUSSION

Mastitis, an inflammation of mammary tissues, is a common disease in humans and animals [26, 27]. *S. aureus*, an important pathogen, frequently causes both types of mastitis, clinical and subclinical, in lactating females [28]. Intramammary infection of *S. aureus* often results in serious tissue destruction and a strong host inflammatory response [29], and most cases cannot be effectively treated. In the work presented here, we successfully created mouse and cell models of *S.aureus* mastitis. Histopathological examination showed mammary gland infiltration by multiple inflammatory cell types and mammary gland structural damage in the *S. aureus* group, similar to what has been observed in previous research [4, 30]. We observed that luteolin obviously relieved *S. aureus*-induced histopathological changes in the mammary gland in a dose-dependent manner.

Pro-inflammatory cytokines, including interleukins and TNF-α, are deemed to initiate the inflammatory reactions in mammary tissues [31, 32]. In the present study, qPCR and ELISA analysis were used to evaluate the mRNA and protein levels of inflammatory cytokines in *S. aureus* mastitis. The expression of TNF-α, IL-6, and IL-1β, which was induced by *S. aureus* infection, were inhibited by luteolin treatment in a dose-dependent manner. TNF-α, IL-6 and IL-1β play important roles in the inflammatory response [33]. TNF-α is an early cytokine, which plays a critical role in the cascade of other pro-inflammatory cytokines and inflammatory mediators [34]. IL-1β which is considered to be a gatekeeper of inflammation plays an essential part in the early inflammatory response [35]. IL-6 is a pleiotropic cytokine involved in the physiology of virtually every organ system [36]. IL-6 production is promptly increased in acute inflammatory responses associated with infection, injury, trauma, and other stress [37]. These cytokines sustain the extracellular and intracellular growth of *S. aureus*, but their overproduction can lead to systemic inflammation, with destructive rather than protective effects on the host [38]. The inhibition of TNF-α, IL-1β, and IL-6 production is helpful for preventing inflammation. According to our results, luteolin exerted anti-inflammatory effects by regulating TNF-α, IL-1β, and IL-6 and by preventing *S. aureus*-induced damage to the mammary tissues and the mammary epithelial cells.

TLR-2 is the major receptor for the immune identification of *S. aureus*, and recent studies have revealed that TLR-4 plays a similar role in the process of *S. aureus* inflammation [39]. In this study, the expression of TLR-2 and TLR-4 were both significantly increased in the *S. aureus* group, but decreased with luteolin treatment, in agreement with previous research [40]. The past work showed that excessive expression of TLR-2 and TLR-4 marked an active inflammatory reaction and triggered the downstream NF-κB signaling pathway [41, 42]. NF-κB p65, a critical regulatory transcription factor, is involved in major inflammatory pathways downstream of TLRs that regulates the expression of many inflammatory genes and the production of cytokines [43]. NF-κB exists in the cytoplasm in an inactive form bound to the IκB inhibitors. The p65 unit separates from IκBα when stimulation occurs and translocates into the nucleus, triggering the transcription of multiple inflammatory genes [23, 44]. The results of the present study showed that *S. aureus*-stimulated phosphorylation of IκBα and p65 was attenuated by luteolin in a dose-dependent manner. Previous research showed the inhibition of NF-κB p65 phosphorylation could reduce inflammatory damage of the mammary gland [45]. Our results revealed that luteolin reduced the production of TNF-α and IL-6 via inhibiting of NF-κB p65 by blocking IκBα degradation and p65 phosphorylation. These results were similar to those of previous studies [4, 30]. The expression levels of TLR-2 and TLR-4 and NF-κB pathway activation further confirmed that luteolin could inhibit inflammation.

MMPs, a family of zinc-dependent endopeptidases, are collectively capable of breaking down most components of the basement membrane and facilitating cell migration [46]. TIMPs are critical endogenous regulators of MMP activity in the tissue, specifically inhibiting MMPs and preserving stromal integrity [47]. Some studies have proved that cytokines promote the expression of MMP-2 and MMP-9 [48, 49]. Moreover, the appearance of TNF-α and IL-6 in the early stage of inflammation indicates that MMP-2 and MMP-9 have a close relationship with the inflammatory reaction. Meanwhile, the invasion of *S. aureus* also leads to increased expression of MMPs. Our results showed that luteolin decreased the levels of MMP-2 and MMP-9 in a dose-dependent manner. In contrast, TIMP-1 and TIMP-2 inhibit MMP-9 and MMP-2, respectively. The levels of TIMP-1 and TIMP-2 were significantly increased in the LAG group in a dose-dependent manner. These findings indicated that luteolin had a protective effect on mammary gland tissues by inhibiting the synthesis of MMPs and increasing TIMPs, which is certainly a novel finding.

The results of this study demonstrate that luteolin had a protective anti-inflammatory effect in the *S. aureus*-infected mouse mammary gland. Luteolin inhibited the expression of TLR-2 and TLR-4 and activation of NF-κB pathway to reduce the pro-inflammatory factor expression and increase the anti-inflammatory cytokines production. In total, our results suggest that luteolin may be a potential prophylaxis or treatment for *S. aureus* mastitis and other inflammatory diseases.

## MATERIALS AND METHODS

### Animals and experimental groups

A total of 60 adult postpartum and lactating BALB/c mice, 6–8 weeks and weighing approximately 25 to 30 g, were used in the present study. These mice were purchased from the Center of Experimental Animals of Wuhan Institute of Biological Products Co. Ltd (Hubei, China). This study was approved by the Huazhong Agricultural University Animal Care and Use Committee in accordance with the Guide for the Care and Use of Laboratory Animals published by the US National Institutes of Health. The mice were housed at 24 ± 1°C, and relative humidity was 40–80%. The mice were housed for 4–6 days to adapt to the environment before the experimentation.

Luteolin (Purity: > 98%) was dissolved in sterile phosphate-buffered saline (PBS) at concentrations of 25, 50, and 100 mg/kg. To establish the mouse model of mastitis, a 100 μl syringe with a 30-gauge blunt needle was used to inoculate 100 μl *S. aureus* (1 × 10^7^ CFU per 10 μl) via teat canal for inducing infection in the mammary gland through both the L4 (on the left) and R4 (on the right) abdominal mammary glands. Sixty lactating mice, 5–7 days after giving birth, were randomly divided into four groups:

(1) The blank control group (BCG): without mastitis or treatment.

(2)The *S. aureus*-stimulated group (*S. aureus*): the mouse model of mastitis without drug treatment.

(3–5)The luteolin administration groups (LAGs): the mouse model of mastitis intraperitoneally administered luteolin at 25, 50, and 100 mg/kg, injected four times at 6, 12, 18, and 24 h after *S.aureus* stimulation for 24 h.

(6) The dexamethasone administration group (DEX): the mice subjected to mastitis by *S.aureus* and intraperitoneally administered dexamethasone. After treatment, mice were euthanized by CO_2_ inhalation, and the mammary gland was harvested and kept at −80°C storage.

### Histopathologic evaluation

The mammary gland tissues for histopathological examination were fixed with 10% buffered formalin, dehydrated with graded alcohol, and made transparent in xylene. After paraffin-imbedding and hematoxylin and eosin (H&E) staining, mammary gland tissues were observed for pathological changes under a light microscope.

### Cell culture and identification

mMECs were extracted from the mammary glands of late pregnancy mice. The shredded mammary tissue was digested with collagenase I and II and pancreatic enzymes for 2 h per gram of tissue. The digestion was terminated with medium containing 10% Fetal bovine serum (FBS) and centrifuged. We subsequently removed the supernatant and added 5 ml medium to a T25 culture flask. The cells were cultured in Dulbecco's Modified Eagle's Medium (DMEM, Hyclone, USA) supplemented with 10% (*v/v*) FBS under humidified air containing 5% CO_2_ at 37°C. The mammary epithelial cells were identified by expression of CK-18, which is a characteristic epithelial cell marker.

### Cell groups

Mammary epithelial cells were seeded in six-well plates. Cells were divided into groups as follows: 1) BCG: cells without any treatment. 2) *S. aureus*-stimulated group (*S. aureus*): the cells were stimulated with *S. aureus* without drug treatment. 3) LAGs: the cells were incubated with 2.5, 5, or 10 μg/ml luteolin. After 1 h of incubation, 1 × 10^6^ CFU/ml *S.aureus* was added into wells and incubated 2 h. 4) Dexamethasone administration group (DEX): the cells were stimulated by *S.aureus* and were administered dexamethasone.

### MTT assay

Logarithmic-phase cells were collected and put into 6 well plates (100 μl each well). After incubation at 37°C and 5% CO_2_, drug was added into the well and 5 repeating groups were set up. After 16–48 h, 20 μl MTT was added to each well. Then, the culture was terminated, and the culture solution was removed. Next, 150 μl dimethyl sulfoxide was added and the 6 well plates were placed on a shaker table for 10 min. The 490 nm absorbance of each well was measured using an enzyme-linked immune detector.

### Enzyme-linked immunosorbent assays

After mammary gland tissue homogenization in PBS and collection of the culture medium, the tissues and the medium were centrifuged. The supernatants were collected to determine the levels of TNF-α, IL-1β and IL-6 by ELISA kits in accordance with the manufacturer's instructions. The attenuance was determined at 450 nm with a microplate reader (Thermo Scientific Multiskan MK3, USA).

### Western blot analysis

The mammary tissue samples and the cells were harvested. Total protein was extracted in 1mL or 200 μl lysis buffer supplemented with protease inhibitor. The protein concentrations were determined using the BCA protein assay kit. For Western blot analysis, an equal amount of protein (50 μg) from each sample was separared on 10% SDS polyacrylamide gels, electrophoretically transferred onto polyvinylidene difluoride (PVDF) membrane, and blocked in 5 % skim milk in tris-buffered –saline with 0.1% Tween-20 (TBST) for 2 h at room temperature. The membranes were hybridized with primary antibodies (1:1000 dilution) at 4°C overnight. Subsequently, the membrane washed with TBST and incubated with secondary antibody (1:4000 dilution) for 1 h at room temperature. Densitometric values of immunoblot signals were developed with the ECL Plus Western Blotting Detection System (Image Quant LAS 4000 mini, USA). β-actin was used as a control.

### Quantitative real-time polymerase chain reaction

The total RNA from mammary gland tissues and cells was extracted using Trizol regent according to the manufacturer's recommendation (Invitrogen, USA). The concentration and purity of the total RNA were measured spectrophotometrically using the 260/280 nm ratio; then the RNA was reverse transcribed as cDNA. The cDNA was diluted fivefold with sterile water and stored at −80°C. The primers used for measuring steady state levels of mRNA are shown in Table 1. The amplification conditions were as follows: 95°C for 10 min, 40 cycles of 95°C for 15s, 60°C for 60s, and 72°C for 60s. The PCR reaction system (20 μl in total) contained 10μl SYBR qPCR Mix (Roche, Basel, Switzerland), 1μl of each primer, 1 μl cDNA, and 7μl nuclease-free water. Expression levels of each target gene were standardized to the corresponding GAPDH threshold cycle (Ct) values using the 2^−ΔΔCt^ comparative method.

### Data analysis

The statistical analysis of the data was performed using the SPSS15.0 statistical software for Windows. The significance was determined via a one-way ANOVA using a significance level of *p* < 0.05. The results are presented as means ± SD. Comparisons between groups were performed with ANOVA followed by Dunnetts test. A *p* value of < 0.05 was considered to be statistically significant.
